# Precise medicine of programmed cell death-1/programmed cell death 1 ligand 1 inhibitor immunotherapy combined radiotherapy for inoperable advanced lung cancer: A protocol for systematic review and meta-analysis

**DOI:** 10.1097/MD.0000000000026367

**Published:** 2021-06-18

**Authors:** Gang Liu, Xiaolan Lv, Yanling Ding, Yongbo Guo

**Affiliations:** a Tourism College, Hainan University, Haikou, Hainan; b Department of Laboratory Medicine, Liuzhou Maternity and Child Healthcare Hospital, Liu zhou, Guangxi; c Pneumology Department, 986 Hospital of People's Liberation Army Air Force, Xi’an, Shaan Xi, P.R. China.

**Keywords:** immunotherapy, lung cancer, programmed cell death-1/programmed cell death 1 ligand 1 inhibitor, precise medicine, radiotherapy, systematic review

## Abstract

**Background:**

Programmed cell death-1/programmed cell death 1 ligand 1 (PD-1/PD-L1) inhibitors are a group of immune checkpoint inhibitors immunotherapy for cancer treatment. These immune checkpoint inhibitors are becoming first-line treatments for several types of cancer. Radiotherapy for cancer is a traditional treatment and the therapeutic effect is not satisfactory due to the side effect of chemotherapeutic drugs. This study aims to evaluate the efficacy and safety of PD1/PD-L1 inhibitor immunotherapy combined chemotherapy for inoperable advanced lung cancer.

**Methods:**

We will utilize PubMed, PubMed Central, EMbase, Medline, CNKI, WAN FANG Database, and Web of Science to screen eligible studies published from January 1, 2015 to December 30, 2020. Two reviewers will extract data and evaluate the risk of bias independently. The quality of the included studies will be evaluated using the RevMan 5.3 software for data analysis.

**Results:**

This review will summarize high-quality evidence of trials to evaluate the precise medicine efficacy and safety of PD1/PD-L1 inhibitor combined radiotherapy for inoperable advanced lung cancer.

**Conclusions:**

The findings of the systematic review will provide scientific evidence of the efficacy and safety of PD1/PD-L1 inhibitor combined radiotherapy for inoperable advanced lung cancer to guide the clinician's drug use.

**Ethics and dissemination:**

Not applicable.

**INPLASY registration number:**

INPLASY202140123.

## 1 Introduction

Lung cancer is the most common tumor in the world, and its morbidity and mortality rank first among all kinds of malignant tumors.^[1,2]^ According to statistics, the number of new cases of lung cancer accounts for 12.9% of all tumor types every year. Lung cancer is a malignant disease occurring in the bronchial mucosa epithelium. And 85% were non-small cell lung cancer (NSCLC).^[3]^ With the deepening of clinical studies on the immune escape mechanism of the tumor, it is found that programmed cell death 1/programmed cell death ligand 1 (PD-1 ligand, PD-L1) can suppress the immune response through immune escape, immunosuppression, and clearance and other mechanisms to enhance the tumor microenvironment on the body's normal immunity resistance effect.^[4]^ Regarding immunosuppressive agents targeting the PD-1/PD-L1 pathway, its efficacy and safety have been confirmed,^[5]^ in large clinical trials of locally advanced maintenance chemotherapy and advanced first-line and second-line treatments in NSCLC,^[6]^ but the domestic clinical reports of anti-PD-1/PD-L1 single treatment combined radiotherapy for inoperable advanced NSCLC are seldom reviewed.

Therefore, this study will evaluate the effectiveness and safety of PD1/PD-L1 inhibitor combined radiotherapy for inoperable advanced lung cancer. The findings of the systematic review will provide scientific evidence and an important reference for clinical decision-making for inoperable advanced lung cancer.

## 2 Methods

### 2.1 Study registration

This protocol of systematic review and meta-analysis has been registered with the International Platform of Registered Systematic Review and Meta-Analysis Protocols (INPLASY) (Inplasy protocol 202140123. https://inplasy.com/inplasy-2021-4-0123/; its registration number is INPLASY202140123).

### 2.2 Ethics

Ethical approval is not necessary because this is a protocol, and the data included in our study are searched from published literature.

### 2.3 Inclusion criteria for study selection

#### 2.3.1 Type of studies

Randomized controlled trials involving PD1/PD-L1 inhibitor combined radiotherapy for inoperable advanced lung cancer will be included. The language will be limited to Chinese and English.

#### 2.3.2 Type of participants

Participants conformed to guidelines for diagnosis and treatment of inoperable advanced lung cancer will be included, regardless of sex, age, and race.

#### 2.3.3 Type of interventions

The control group was treated with radiotherapy alone, and the treatment group was treated with PD1/PD-L1 inhibitor combined radiotherapy, regardless of the dosages.

#### 2.3.4 Type of outcome measures

The main outcome indicator was the clinical efficiency, and the efficacy was evaluated according to overall response rate (ORR) and disease control rate (DCR) for immune checkpoint inhibitors, progression-free survival rates (PFS) for lung cancer, and a significant improvement in PFS was observed in lung cancer. Additional outcomes included white blood cell count and incidence of adverse events, with a combined hazard ratio and 95% confidence interval.

#### 2.3.5 Data source

The following databases will be searched from January 1, 2015 to December 30, 2020: The Cochrane Library, PubMed, EMBASE, Web of Science, China National Knowledge Infrastructure, and Wanfang Data. All the English and Chinese publications are searched without any restriction of countries.

### 2.4 Search strategy and study selection

#### 2.4.1 Search strategy

Different search strategies will be applied according to the requirements of different databases. Also, references can be searched, and other resources, such as conferences and books, can be used to supplement relevant literature. Search strategy was showed in Annex 1, Supplemental Digital Content.

#### 2.4.2 Selection of studies

**Figure 1:**
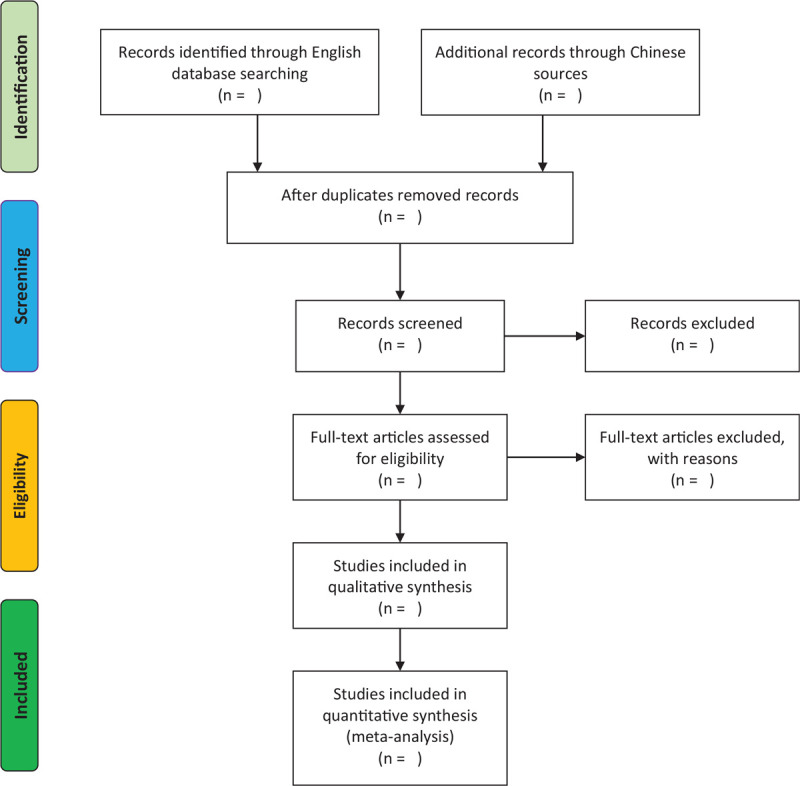
Flow chart of screening literature.

Two authors will independently eliminate relatively unrelated literature by scanning titles, abstracts. Duplicate articles will be removed and references will be managed by EndNote X9 software (Clarivate, US). When there is a dispute, the 2 reviewers (XL and YD) will read the full text. The screen flow is described in Fig. 1. Any disagreement will be solved by discussion.

### 2.5 Data extraction and analysis

#### 2.5.1 Data extraction

Data will be extracted by 2 reviewers independently (XL and YD). Any disagreement will be resolved through discussion until consensus is reached or by a third author. The following data will be extracted: general information: author, year of publication, the country of the study conducted; database. Participant characteristics, including age, sex, stage of lung cancer, therapeutic method; total number of patients included in the study; Intervention details: doses of radiotherapy, dose of anti-PD-1/PD-L1, and the time of application. Adverse effect and quality assessment tools. A characteristics of included reviews were detailed in Table 1.

**Table 1:** Characteristics of included reviews.

Author	Year	Country	Database	Participant characteristics	Intervention	Outcomes	Adverse effect	Quality assessment tools
								

#### 2.5.2 Strategy for data synthesis

The analysis and synthesis of data will be conducted by RevMan 5.3 software (Copenhagen, The Nordic Cochrane Center, the Cochrane Collaboration). Risk ratio with a 95% confidence interval (CI) will be used to determine dichotomous data or standardized mean differences (95%) CI will be used to analyze the continuous data.

#### 2.5.3 Risk of bias assessment

The characteristic included random sequence generation, participant blinding, outcome assessor blinding, and other generic sources of bias, attrition, and exclusions. The methodological quality of randomized controlled trials will be assessed by Cochrane risk of bias.

#### 2.5.4 Subgroup analysis

To seek whether there is a possible causes of heterogeneity, we will perform subgroup analysis if there are a sufficient number of literatures.

#### 2.5.5 Sensibility analysis

In order to make sure the results are reliable, we will perform sensitivity analysis to eliminate the impact of low-quality literatures after validation of inputted data and subgroup analysis. But, if all included literatures are at high risk of bias, we will not perform sensitivity analysis.

## 3 Discussion

PD-1/PD-L1 immunotherapy combined with radiotherapy is advanced treatment for lung cancer as precise medicine, which can achieve better effect, mostly it is because, radiotherapy can promote the immune system to produce remote effect to eliminate distant tumor (abscopal effect). In our previous department study,^[7]^ we used immune cell combined with radiotherapy to achieve good cancer treatment effect. Thus we summarize a system review and meta-analysis to conclude a perspective sight to guide clinical use.

PD-1/PD-L1 signaling pathway in a variety of tumor cells and tumor microenvironments. The expression of PD-1/PD-L1 can be effectively blocked by anti-PD-1/PD-L1 monoclonal antibody. This signaling pathway activates the lymphatic system, resulting in powerful anti-tumor activity. The cell-killing effect has a significant potential to improve the efficacy of NSCLC.^[8–10]^ Nivolumab is a human PD-1 immune checkpoint inhibitor antibody, which can be blocked.^[11]^ Disrupting PD-1 mediated signaling pathway and restoring tumor immunity in lung cancer in recent year. Satisfactory progress has been made in clinical application.^[11,12]^ For example, Gauvain et al^[13]^ used it in patients with NSCLC brain metastases, the results showed an intracerebral control rate of 51% and safety is acceptable.

Currently, the treatment of advanced NSCLC is based on platinum-containing drugs. However, based on radiotherapy, changes are happening with each passing day.PD-1/ PD-L1 inhibitors have led to the development of a new treatment for NSCLC. A brand new phase. PD-1/PD-L1 inhibitors work by blocking tumor cells. The interaction between PD-L1 expression and PD-1 expression in T cells is regulated. The immune response of the body can restore the proliferation and activation of CD4+T/CD8+ cells. To inhibit the escape, proliferation, and metastasis of tumor cells.^[14,15]^ Single drug first-line treatment with PD-1/PD-L1 inhibitors was obtained in advanced NSCLC that achieved better efficacy in the Keynote024 clinical trial.^[16]^ Similar results were also shown in the BIRCH clinical trial with natalizumab which has a better curative effect than radiotherapy.^[17]^

The cytotoxic effect of chemotherapeutic drugs directly kills tumor cells, by inducing the immunogenic death of tumor cells and promoting tumor correlation. The formation of sex cells and other functions change the local tumor immune microloop.^[18]^ In the clinical trial,^[19,20]^ the immune system was analyzed. Epidemic combined with targeted radiotherapy in first-line advanced non-squamous cell carcinoma (NSCLC). The results showed that atalizumab was combined with the antiangiogenic and radiotherapy.

At the same time, the types and extent of adverse reactions, as well as their control and management, are critical. A very important link in bed work. In the case of adverse reactions, combined treatment, a good response should be treated with a suspension of administration and consideration of glucocorticoid, which can be controlled,^[17,21]^ and the treatment principle for more serious adverse reactions are as follows: early detection, early assessment, and early treatment.^[22,23]^ In summary, this study will be comprehensive to evaluate the efficacy and safety of PD1/PD-L1 inhibitor combined radiotherapy for inoperable advanced lung cancer, which may provide the direction that PD-1/PD-L1 inhibitors combined with radiotherapy can be used as first-line therapy for inoperable advanced lung cancer.

## Author contributions

**Conceptualization:** Gang Liu, Yongbo Guo.

**Data curation:** Gang Liu, Yongbo Guo.

**Formal analysis:** Xiaolan Lv, Yanling Ding.

**Funding acquisition:** Gang Liu, Xiaolan Lv, Yanling Ding.

**Investigation:** Yanling Ding, Yongbo Guo.

**Methodology:** Xiaolan Lv, Yongbo Guo, Gang Liu.

**Project administration:** Xiaolan Lv, Yanling Ding, Yongbo Guo.

**Resources:** Xiaolan Lv, Yongbo Guo, Gang Liu.

**Software:** Yongbo Guo, Gang Liu.

**Writing – original draft:** Gang Liu, Yongbo Guo.

**Writing – review & editing:** Gang Liu, Yongbo Guo.

## Supplementary Material

Supplemental Digital Content
