# Supplementary material for: Precise medicine of programmed cell death-1/programmed cell death 1 ligand 1 inhibitor immunotherapy combined radiotherapy for inoperable advanced lung cancer: A protocol for systematic review and meta-analysis
Source: Medicine (Baltimore). 2021 Jun 18;100(24):e26367. doi: 10.1097/MD.0000000000026367 (PMC8213331; doi:10.1097/MD.0000000000026367)
Supplement: Supplemental Digital Content [file medi-100-e26367-s001.doc]

**Appendix 1 Search strategy**

(1) PubMed

| **NO.** | **Search strategy** | **items** |
| --- | --- | --- |
| #1 | " **inoperable advanced lung cancer** "[Mesh] |  |
| #2 | (**inoperable advanced lung cancer** [Title/Abstract]) OR **lung cancer** [Title/Abstract] |  |
| #3 | #1OR#2 |  |
| #4 | ("**radiotherapy** "[Mesh]) OR ("**immunotherapy** "[Mesh]) |  |
| #5 | ((**radiotherapy** *[Title/Abstract]) OR **PD1/PD-L1 inhibitor** [Title/Abstract]) OR **precise medicine**[Title/Abstract] |  |
| #6 | #4 OR #5 |  |
| #7 | "Meta-Analysis" [Publication Type] OR "Meta-Analysis as Topic"[Mesh] |  |
| #8 | (Meta analys*[Title/Abstract]) OR Systematic review*[Title/Abstract] |  |
| #9 | #7 OR #8 |  |
| #10 | #3 AND #6 AND #9 |  |

(2) Embase

| **NO.** | **Search strategy** | **items** |
| --- | --- | --- |
| #1 | ' **inoperable advanced lung cancer** ' /exp |  |
| #2 | ' **inoperable advanced lung cancer** ':ab,ti OR ' **lung cancer** ':ab,ti |  |
| #3 | #1 OR #2 |  |
| #4 | ' **radiotherapy** '/exp |  |
| #5 | **radiotherapy** *:ab,ti OR **PD1/PD-L1 inhibitor**:ab,ti OR **precise medicine** ':ab,ti |  |
| #6 | #4 OR #5 |  |
| #7 | 'meta analysis'/exp |  |
| #8 | 'meta analys*':ab,ti OR 'systematic review*':ab,ti |  |
| #9 | #7 OR #8 |  |
| #10 | #3 OR #6 OR #9 |  |

(3) Cochrane Library

| **NO.** | **Search strategy** | **items** |
| --- | --- | --- |
| #1 | MeSH descriptor: [**inoperable advanced lung cancer**] explode all trees |  |
| #2 | ("**inoperable advanced lung cancer** "):ti,ab,kw AND ("**lung cancer** "):ti,ab,kw |  |
| #3 | #1 OR #2 |  |
| #4 | MeSH descriptor: [**radiotherapy**] explode all trees |  |
| #5 | (**radiotherapy** *):ti,ab,kw OR (**PD1/PD-L1 inhibitor**):ti,ab,kw OR ("**precise medicine** "):ti,ab,kw |  |
| #6 | #4 OR #5 |  |
| #7 | MeSH descriptor: [Meta-Analysis] explode all trees |  |
| #8 | ("Meta analys*"):ti,ab,kw OR ("Systematic review*"):ti,ab,kw |  |
| #9 | #7 OR #8 |  |
| #10 | #3 AND #6 AND #9 |  |

(4) Chinese databases

| **Databases** | **Search strategy** | **items** |
| --- | --- | --- |
| CNKI | (不可手术肺癌 OR 肺癌OR AD) AND (放疗OR免疫治疗OR PD1/PD-L1 抑制剂OR精准治疗) AND (系统评价OR系统综述OR荟萃分析OR meta分析OR元分析) |  |
| WANFANG DATA |  |
